# Engineering education in the wake of hurricane Katrina

**DOI:** 10.1186/1754-1611-1-6

**Published:** 2007-10-10

**Authors:** Marybeth Lima

**Affiliations:** 1 Department of Biological & Agricultural Engineering, Louisiana State University, LA, USA

## Abstract

Living through hurricane Katrina and its aftermath and reflecting on these experiences from technical and non-technical standpoints has led me to reconsider my thoughts and philosophy on engineering education. I present three ideas regarding engineering education pedagogy that I believe will prepare future engineers for problem-solving in an increasingly complex world. They are (1) we must practice radical (to the root) engineering, (2) we must illustrate connections between engineering and public policy, and (3) we will join the charge to find sustainable solutions to problems. Ideas for bringing each of these concepts into engineering curricula through methods such as case study, practicing broad information gathering and data interpretation, and other methods inside and outside the classroom, are discussed. I believe that the consequences of not considering the root issues of problems to be solved, and of not including policy and sustainability considerations when problems to be solved are framed will lead our profession toward well meaning but insufficient utility. Hurricane Katrina convinced me that we must do better as educators to prepare our students for engineering for a sustainable world.

## Background

Hurricane Katrina struck the gulf coast on August 29, 2005. This natural disaster catastrophically affected the U.S. gulf states and negatively impacted the entire country. My place of residence, Baton Rouge, Louisiana, survived hurricane Katrina with serious but non-catastrophic damage because the edge of the 40 mile wide eye of the storm passed 90 miles to the east of the city.

I experienced many events analytically and from an engineering perspective, for example, the doubling of our city population, traffic gridlock, loss of communication (two days), loss of power (three days), and a gas shortage (none for five days, shortage for five weeks). Volunteering in the aftermath of Katrina provided many more experiences that were primarily emotional. I volunteered at the University of New Orleans satellite office that was established on the Louisiana State University (LSU) campus just after the hurricane. Employees were supposed to call in with contact information and their evacuation location; about half the calls I fielded were from relatives of the employees who were trying to ensure that their loved ones were okay. On the rare occasions that a loved one's relative had already contacted us, I could inform the caller that the person was safe, but I wasn't allowed to provide the person's phone number for liability reasons.

School classrooms and boy and girl scout troops from all over the country sent school supplies for displaced children; while unpacking these supplies, our public school distribution team read heartfelt words of hope and kindness that frequently brought us to tears.

The LSU AgCenter livestock barn, the Parker Coliseum, was turned into a holding area for pets evacuated from the storm. Of all my experiences, the wall of animals seeking people was the most poignant. While staring at approximately 150 Polaroid pictures of animals replete with their names and tags and the words OWNER MISSING printed on every photograph, I realized that I never would have conceived of a wall like that.

Reflecting on my experiences while living through hurricane Katrina made me recognize that there are better ways to teach engineering and better ways to engage with our communities. My teaching philosophy changed after hurricane Katrina in two ways: (1) My understanding of sustainability and policy issues was deepened by an experiential perspective (which was purely academic before Katrina), and (2) because I realized that although I strive to teach students to see the big picture, in the context of Katrina (particularly the wall of animals), I completely missed it myself.

In terms of applying the lessons learned from Katrina to improve the education of our students and to further our profession, I have three major ideas:

1. We must practice radical (to the root) engineering

2. We must illustrate connections between engineering and public policy

3. We will join the charge to find sustainable solutions to problems

I will describe each of these concepts in more detail in the sections that follow.

### 1. We must practice radical (to the root) engineering

The word radical has multiple and disparate meanings in science, mathematics, and politics. Radical means "of or going to the root or origin" [1]. Practicing "to the root" engineering means that we address immediate problems through engineering problem-solving and design, but that we also address the root issues that caused the problem in the first place. For example, we can address the immediate engineering problems in New Orleans that were caused as a result of hurricane Katrina, but we can also direct efforts at the root issues that caused Katrina to be so devastating to New Orleans in the first place (destruction of wetlands, land subsidence, and global warming).

In an engineering education context, the concept of radical engineering translates into teaching students to consider engineering from short and long term standpoints, with the short term focus on addressing immediate issues and the long term focus on addressing the root causes of issues.

A factor that complicates short and long term thinking within engineering is that the rate of change of technology and the rate of information creation is increasing ([2-4]). The total amount of technical information produced worldwide doubles every ten to fifteen years. Students will need to respond quickly to changes and will need to realize how the increasing rate of change impacts radical problem-solving. One strategy that has proved invaluable to teach students to find, monitor, and interpret information is having a librarian teach and work with students on data mining and navigation.

One way to illustrate the way in which technical information evolves as a function of time is to study the changing nature of engineering standards. For example, professional engineering licensure requires the engineer to complete continuing education hours to ensure, among other things, that the engineer keeps current with engineering standards that s/he uses on a regular or occasional basis. The engineer only needs to pass the professional engineer's exam once to be licensed. To be a certified playground safety inspector (CPSI), a candidate must be proficient with safety guidelines produced by the Consumer Product Safety Commission (CPSC) and technical standards published by ASTM. A CPSI must re-certify every three years by taking a review course AND the national exam. The rationale is that CPSC guidelines and ASTM standards can and do change drastically in response to very few (even one) child injury or fatality. Future engineers must be cognizant of the dynamic nature of engineering standards.

Another example to illustrate the changing nature of engineering standards and data is to consider the aviation industry. In 2003, US Airways Express Flight 5481 crashed just after take off in Charlotte, NC. The cause of the crash was loss of pitch control, and several factors contributed to this cause. One factor was the weight of passengers and bags on the plane [5].

US Airways was using the average weight method to estimate the plane's weight. This method assumed that passengers on a flight are 60% men and 40% women, and was using average weight data for adult men and women that was approximately twenty-five years old. The weight of an average man in the USA has increased from 166 pounds in 1960 to 191 pounds in 1999, while the average weight of a woman has increased from 140 pounds in 1960 to 164 pounds in 1999 [6]. The US Airways flight had 16 men and 3 women on board. The weight of each person on board was underestimated by 21 pounds and the weight of each bag was underestimated by 6 pounds. Although this extra weight may seem like a small factor, the weight of people and their bags accounts for almost 25% of the total weight of a commuter flight like Flight 5481.

The FAA responded to this plane crash with a new standard method in notice N8400.40; it states that planes that carry 10–19 passengers must use actual weights vs. average weights. Case studies such as these can be used to illustrate to students the importance of using recent data in engineering design and the evolving of engineering standards in response to a tragic accident like a plane crash.

Enlisting the assistance of a librarian and teaching students about the changing nature of engineering standards are two solid educational strategies for thinking about problem-solving from near term (immediate) and far term (consequential) perspectives.

Another strategy is to teach students to focus on "what will be," which may involve asking students to extrapolate or model design parameters based on current and projected rates of change. We already use such projections with the design of water control structures, whose parameters are based on the probabilities of storms occurring (for example, a 25 year, 24 hour storm). It is instructive to have students answer the following (geographically specific) questions: What is a 100 year storm now? What will a 100 year storm be in 25 years? In 100 years?

After hurricane Katrina, there was much focus on the levies that failed, which resulted in the flooding of 80% of the city. The Netherlands were frequently cited as having a superior flood control system that was designed using 10,000 year storm data. In the United States, water control structure textbook design data stops at 100 years. It is as if those of us that practice engineering have not given thought to a situation in which we would need to use storm data more rigorous than this. It would be difficult for our students to conceive of something that they have never seen – students would probably not ever consider why >100 year storm data would be used because the books stop at 100 years. I believe that it is critical for engineering educators to illustrate to students the extremes of the design process, and when and why extreme data are sometimes used. I had never conceived of using greater than 100 year storm data before the hurricane (even though Katrina was not a 100 year storm) because I never saw the information tabulated in a design manual. This approach is akin to being "asleep at the wheel." Ultimately, students must be exposed to "scales" of data or to models when data are not available that will enable them to see additional perspectives and approaches to problem-solving and design.

Radical engineering involves students thinking about immediate and consequential issues in problem-solving, keeping current with information and engineering standards, being able to specify appropriate design parameters based on increasing rates of change and increasing complexity, and having the driving force in the design process be addressing root causes of problems with a focus on the future.

### 2. We must illustrate connections between engineering and public policy

The engineering science-based approach which forms the basis of most engineering curricula in the United States is heavily geared toward cultivating problem-solving and technical skills. Future engineers must be taught technical skills and how they are applied in the public sphere. I believe that applications of engineering in the public sphere and in public policy will be a critical skill set for the future engineer given the increase in the rate of change of technology and information generation, and the convergence of disparate disciplines that is required to solve increasingly complex problems.

Public policy drives which technology is adopted and focused on, and these types of decisions are heavily influenced by the business community. In terms of who is making public policy, consider the following: in 2003–2004, the U.S. House of Representatives had five members with engineering degrees and eight members with science degrees, out of a total of 435 members [3]. Although politicians have technical advisors on their staffs and advisory boards such as the National Academies of Science, the National Academy of Engineering, and the Institute of Medicine to guide them, the overwhelming majority of our elected decision makers do not possess technical expertise.

Many engineering educators tend to steer clear of political and policy issues because they believe that we should teach students to be objective, and political and policy issues are not necessarily objective. I contend that as educators, we should continue to teach engineers to be objective, but we should also teach the political process (or at the very least, work closely with those who do to make this an integral part of the curriculum). Essentially everything we do professionally is affected by politics, whether we are comfortable with this concept or not.

If we apply these ideas to post-Katrina New Orleans (coupled with near and far term radical engineering thinking), current political and policy discussions involve answering the following questions:

• Should we re-build New Orleans?

• Should we re-build parts of New Orleans and if so, which parts?

• Should we address why New Orleans was vulnerable in the first place?

Answering these questions requires understanding and knowledge of technical and non-technical factors. In terms of tackling the third question, we know that New Orleans is and has been increasingly vulnerable to hurricanes because of land subsidence, destruction (development) of wetlands, and increasing water (sea) levels. According to leading scientists, the latter cause is due at least in part to global warming ([7-10]). Global warming is a political issue as well as a technical one. I contend that a citizen's current position on global warming is based more on political party affiliation than analysis of scientific studies. I further contend that this situation is occurring because politicians are leading the discussion and debate on global warming and are setting policy as a result. If engineers are taught to be objective and to not engage in the policy or political process, then very few will step into policy and political arenas to share their critical technical and problem-solving skills and perspectives. Engineers must pay attention to the national discussion (alarmist, tree hugging liberals versus money grubbing, heartless conservatives) but must also think beyond the national discussion (when and how will New Orleans be re-built in a culturally appropriate manner).

Currently, most of our engineering students do not have the skills to analyze or dissect non-technical arguments and would avoid, ignore, dismiss as being beneath their intellect, or shy away from such discussion or analysis. The consequences of the disconnect between engineering and public policy in our engineering curricula could be dire, both in the context of this example and in a broader, societal context.

Consider the following scenario with respect to global warming: currently, 43% of the U.S. population lives within 100 kilometers of a coastline. Projections are that Manhattan and the Florida Everglades will be under water in the year 2100, and that Baton Rouge will be beach front property in the year 2500. Shall we cede Manhattan to the Atlantic in 93 years? Shall we build, re-build, and re-develop land affected by hurricanes, tornadoes, and earthquakes, and if so, how much? The answers to these and other questions should not be dictated without extensive scientific, social, and engineering input; engineers must play a larger role in the national discussion to help shape policy.

### 3. We will join the charge to find sustainable solutions to problems

The chaos that ensued in the U.S. gulf states after hurricane Katrina caused me to think more deeply about sustainability issues which could be more fully addressed by the engineering profession if students and young professionals were exposed to the connections between engineering and policy.

For example, today the citizens of the United States comprise 5% of the world's population and use 30% of the world's energy. This situation is unsustainable and is compounded by the fact that the available, capable technology of renewable energy is ahead of implementation policy for a myriad of non-technical reasons. Other nations, most notably China and India, are following the U.S. lead and its unsustainable, non-renewable habits.

When the Russians launched the satellite known as Sputnik in 1957 and took the world lead in space exploration, the United States responded by strongly encouraging an entire generation of citizens to study science and engineering to help the United States to re-gain the world lead in space exploration.

I would like to issue a new sustainability edict that engineers and other professions can aspire to: that fifty years from now there be a proportional relationship between population size and resource utilization. The U.S., as 5% of the world's population, should use 5% of the world's energy. To meet this goal, the majority of this energy must come from renewable sources.

I would like to think that the U.S. is the kind of society that can respond to a moral imperative like being sustainable with more vigor than it responded as a nation to the call for scientists and engineers in the wake of the Russians and Sputnik.

Engineering educators can contribute significantly to sustainability efforts that are already existent on their college campuses or can help launch such efforts for their campuses. The United Nations established 2005–2014 as the decade for education on sustainable development (resolution 57/254). Numerous institutions of higher education have responded to this call; college presidents can (and many have) sign(ed) a letter of commitment to make their campuses green through programs like green building design and construction, Leadership in Energy and Environmental Design (LEED) certification, and zero net energy emissions. More than 35 college campuses have already joined this initiative, including Harvard University, Penn State University, and the University of British Columbia [11].

I believe that engineers have a critical role to play in advancing sustainability concepts that are critical to the future survival of our planet and its inhabitants. Engineers will be in a position to use their problem-framing, problem-solving, and critical thinking skills if they practice radical engineering, and if they understand and embrace the connections between engineering and public policy. As engineering educators, we can put our students on this path. For tips on teaching methods with the ideas advanced in this paper, see the section entitled "teaching tips for engineering educators."

#### Teaching tips for engineering educators

You can address short and long term thinking issues through your teaching such that you are teaching "in the now" and teaching toward a sustainable future. The following list of tips is intended to act as a starting point for preparing students for the dynamic environment in which they will be living and working.

##### Teaching suggestions

• Stress to students that there is very little that is constant. This concept can be illustrated using the evolving nature of engineering standards.

• Stress to students that experimental data is not an absolute measure, but is a function of numerous factors that may change the data significantly. Students should know where data came from, under what conditions it was collected, limitations of the data, and if, when, and how to use it (metadata).

• Teach students to find and keep up with new information. You may need to enlist the expertise of a librarian to do this topic justice.

• Integrate sustainability into engineering concepts and discussions. For example, when discussing concepts like pumping power, include examples on renewable power systems.

• Direct students to sustainability efforts already underway (for example, the sustainable campus initiative). Encourage and advise student groups to complete sustainable designs that have a direct impact on the local university and/or community.

##### In class activities

• For the first five minutes of class, play pertinent audio clips from National Public Radio, which has a multitude of stories on science, technology, and policy that are archived online [12]. After playing a story, facilitate a discussion on technical sustainability, or engineering and public policy.

• Students must find popular press articles on a sustainability/policy subject, do a two minute presentation on the article, and have a three minute class discussion immediately afterward.

• When planning field trips, visit departments of public works, or city and state government agencies that handle technical matters within the local community

• When choosing guest speakers, pick science, technology and/or policy advisors, lobbyists, or government employees that involve liaisons between the community and technology.

##### Out of class activities

• Encourage internships in local, state, or federal government that concern public policy and technology as much as you push traditional technical outlets like research experiences for undergraduates or employment at engineering companies.

• Encourage a group of students to write a proposal to the Environmental Protection Agency for the P3 (people, prosperity, and the planet) national student design competition [13]. Serve as their faculty advisor! Part of this competition involves the students displaying their funded project at the national sustainable design exposition on the national mall.

• Share policy related job and career options with students; engineering graduates can be technical advisors for politicians, lobbyists, private or public sector employees that liaison with community and technology, or can run for political office themselves.

## Conclusion

Former president of the National Academy of Engineering William Wulf stated that the biggest changes in the quality of human life between 1899 and 1999 were due to engineering advances, primarily in sanitation [14]. I would like to extend Wulf's thinking into the future as follows: what will we look like in 2099? Although I am not sure of the answers, I know that they will depend on how this question is framed and analyzed. Hurricane Katrina convinced me that we must do better as educators to prepare our students for engineering for a sustainable world. I believe that we can accomplish this goal by sharing the profession of engineering as it is practiced presently, but also with an eye toward the future. I strongly believe that we need to teach (1) information literacy and how to keep up with and ahead of information; (2) how to consider problem framing and problem-solving from short and long term standpoints, (3) the connections between problem-solving and policy, and (4) sustainability concepts, for example, 5 for 5 in 50. Teaching these four major ideas will enhance the biological engineering discipline by enabling us to continue to solve problems at the intersection of biology and engineering in meaningful ways.
